# Medical Image Fusion Based on Rolling Guidance Filter and Spiking Cortical Model

**DOI:** 10.1155/2015/156043

**Published:** 2015-06-03

**Authors:** Liu Shuaiqi, Zhao Jie, Shi Mingzhu

**Affiliations:** ^1^College of Electronic and Information Engineering, Hebei University, Baoding, Hebei 071002, China; ^2^Key Laboratory of Digital Medical Engineering of Hebei Province, Baoding, Hebei 071002, China; ^3^College of Electronic and Communication Engineering, Tianjin Normal University, Tianjin 300387, China

## Abstract

Medical image fusion plays an important role in diagnosis and treatment of diseases such as image-guided radiotherapy and surgery. Although numerous medical image fusion methods have been proposed, most of these approaches are sensitive to the noise and usually lead to fusion image distortion, and image information loss. Furthermore, they lack universality when dealing with different kinds of medical images. In this paper, we propose a new medical image fusion to overcome the aforementioned issues of the existing methods. It is achieved by combining with rolling guidance filter (RGF) and spiking cortical model (SCM). Firstly, saliency of medical images can be captured by RGF. Secondly, a self-adaptive threshold of SCM is gained by utilizing the mean and variance of the source images. Finally, fused image can be gotten by SCM motivated by RGF coefficients. Experimental results show that the proposed method is superior to other current popular ones in both subjectively visual performance and objective criteria.

## 1. Introduction

Multimodal medical image fusion is a hot research topic and drives a lot of attention for increasing demands for diagnosis and treatment of diseases. There are various modalities of medical images today such as computed tomography (CT), magnetic resonance angiography (MRA), magnetic resonance imaging (MRI), and functional MRI (fMRI) [1]. Different modality medical images can reflect different information of human organs such as CT can only provide dense structures like bones and implants with less distortion, while MR can provide normal and pathological soft tissues information. It is really helpful to doctor by combining complimentary features of different imaging modalities into one fused image. For example, MRI/CT imaging can be combined for diagnosis and treatment planning [2, 3].

This paper focuses on the pixel level medical image fusion technology. Up to now, a lot of medical image fusion algorithms have been proposed. Examples include principal component analysis fusion algorithm (PCA) [4], guided filtering fusion algorithm (GFF) [5], medical image fusion algorithm based on wavelet in [6], fusion algorithm based on Contourlet transform (CT) in [7], fusion algorithms based on nonsubsampled Contourlet transform (NSCT) in [8], fusion algorithm based on Ripplet in [9], and fusion algorithm based on Shearlet and PCNN in [10], and so on. Although these methods produce high-quality images, they also will lead to loss of information and pixel distortion due to nonlinear operations of fusion rules and blocky artifacts [11]. To address these problems, Wang et al. proposed a new medical fusion method based on SCM in [11], which can get much better fusion effects; but, in their method, the parameters of SCM are fixed to some constants which will obviously not be widely applicable to all kinds of medical image fusion. Although the gray values of images can be used as the input of SCM like Wang's method, they are more sensitive to environment than the edge information [12].

In our paper, these disadvantages are overcome by using RGF and adaptive threshold in SCM. RGF is an edge aware filtering, and it can remove the small texture of images without blurring the image edge [13]. Therefore, in this paper, RGF is used to extract the saliency (edge information); and then, the coefficients of RGF are normalized and taken as the stimuli of the SCM. In order to be widely applied to all kinds of medical image fusion, adaptive threshold of SCM is proposed.

This paper is organized as follows. In Section 2, we give a brief review of RGF and SCM. In Section 3, we give the steps of the new image fusion algorithm. In Section 4, we demonstrate the experimental results of the proposed method and the comparisons with other typical fusion methods; and, in the last section, we explore some conclusions.

## 2. Rolling Guidance Filter and Spiking Cortical Model

### 2.1. Rolling Guidance Filter

**Figure 1:**
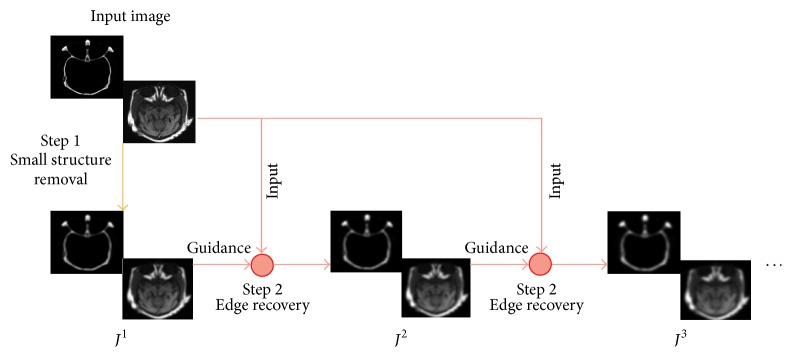
Flow chart of RGF. It contains two steps, respectively, for small structure removal and edge recovery. Edge recovery is an iterative process. The final result is obtained in 3–5 iterations.

Zhang et al. [13] proposed a new framework called RGF to filter images based on a rolling guidance with the complete control of detail smoothing under a scale measure. Compared to other edge preserving filters, RGF is implemented iteratively, which has a fast convergence property. It is simple and fast and also easy to understand. RGF can preserve large-scale structures automatically, where small structure removal and edge recovery are two main steps in RGF; see Figure 1 [13].

Firstly, Gaussian filter is used to remove the small structure. *I* denotes the input image and *G* denotes the output image. *σ*_*s*_ denotes the standard deviation of Gaussian filter. **p** and **q** are the indexes of pixel coordinates in the image. The filter is as follows:

(1) $$\begin{array}{c} G\left(\;p\;\right)=\frac{1}{K_{p}}\underset{q\in N\left(p\right)}{\sum }\exp\left(\;-\frac{{\left\|p-q\right\|}^{2}}{2\sigma_{s}^{2}}\;\right)I\left(\;q\;\right), \end{array}$$

where *K*_**p**_ = ∑_**q**∈*N*(**p**)_exp(−‖**p** − **q**‖^2^/2*σ*_*s*_^2^) is for normalization and *N*(**p**) denotes the set of pixels in the windows of Gaussian filter whose center is at **p**.

Secondly, a joint bilateral filter is used to recover the edge iteratively. Initially, *J* ^1^ is set as the output of the Gaussian filtering. *J* ^*t*+1^ is the output of the *t*th iteration of joint bilateral filtering with the input *I* and *J* ^*t*^. Consider

(2) Jt+1p=1Kp·∑q∈Npexp⁡−p−q22σs2−Jtp−Jtq22σr2Iq,

where *K*_**p**_ = ∑_**q**∈*N*(**p**)_exp(−‖**p** − **q**‖^2^/2*σ*_*s*_^2^ − ‖*J* ^*t*^(**p**) − *J* ^*t*^(**q**)‖^2^/2*σ*_*r*_^2^) is for normalization. *I* denotes the same input image in (2). *σ*_*r*_ controls the range weights.

Finally, two main steps in RGF can be combined into one by starting rolling guidance simply from a constant-value image. In (2), if we set all values in *J* ^*t*^ to a constant *C*, that is, ∀**p**, *J* ^*t*^ = *C*, it updates to *J* ^*t*+1^(**p**) = (1/*K*_**p**_)∑_**q**∈*N*(**p**)_exp(−‖**p** − **q**‖^2^/2*σ*_*s*_^2^)*I*(**q**); the new form is exactly the same as (2).

From Figure 1, we can see that the small structure in medical images is removed by RGF. RGF can remove small-scale structures while preserving other content and is parallel in terms of importance to previous edge-preserving filters. It enlists the power of distinguishing between structures in terms of scales without knowing the exact form (or model) of texture, details, or noise.

### 2.2. Spiking Cortical Model

**Figure 2:**
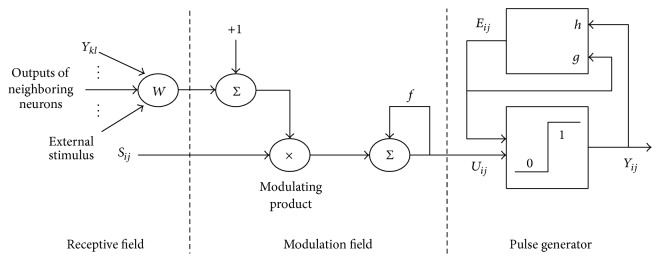
SCM model. The image matrix can be input as external stimulus of SCM.

The SCM [12] is derived from Eckhorn's model and it conforms to the physiological characteristic of human visual neural system. In fact, Wang's method [11] provides an effective means for fusion of the different kinds of medical images. In the spiking cortical model, each neuron consists of three parts: feeding and linking field, modulating product, and pulse generator; see Figure 2.

In the following expressions, the indexes *i* and *j* refer to the pixel location in the image, *k* and *l* refer to the locations of its neighboring pixels, and *n* denotes the current iteration times. The receiving and linking field and modulating product are given by

(3) $$\begin{array}{c} U_{ij}\left(\;n\;\right)=fU_{ij}\left(\;n-1\;\right)+S_{ij}\underset{kl}{\sum }W_{ijkl}Y_{kl}\left(\;n-1\;\right)+S_{ij}, \end{array}$$

where *U*_*i*,*j*_(*n*) is the internal activity and *f* is the attenuation coefficient of *U*_*i*,*j*_(*n*). *S*_*i*,*j*_ is the external stimulus. *W*_*ijkl*_ is the synaptic linking weight and *Y*_*ij*_(*n* − 1) is the previous output pulse.

The pulse generator determines the firing events in the model in (4). *Y*_*ij*_ depends on the internal activity and threshold. Consider

(4) $$\begin{array}{c} Y_{ij}\left(\;n\;\right)=\left\{\begin{array}{cc} 1 & \text{if }\frac{1}{\left(1+\exp\left(U_{ij}\left(n\right)-E_{ij}\left(n\right)\right)\right)}>0.5\\ 0 & \text{otherwise.} \end{array}\right. \end{array}$$

The dynamic threshold of the neuron is defined as

(5) $$\begin{array}{c} E_{ij}^{}\left(\;n\;\right)=gE_{ij}^{}\left(\;n-1\;\right)+hY_{ij}\left(\;n-1\;\right), \end{array}$$

where *g* denotes the attenuation coefficient and *h* denotes the threshold magnitude coefficient. Normally, the size of internal activity matrix *U*_*i*,*j*_(0) is the same as the external stimulus matrix, and *U*_*i*,*j*_(0) is always initialized to zero matrices; and the image matrix *I* can be input as external stimulus of SCM; that is, *S*_*i*,*j*_ = *I*_*i*,*j*_. However, the external stimulus of SCM in this paper is replaced by RGF coefficients of image.

In our paper, we find that the expectation and variance of the sources images can be used to calculate threshold *h* which can reach better fusion results. The adaptive threshold *h* is defined as

(6) $$\begin{array}{c} h=\frac{\sum_{i}\text{mean}\left(I_{i}\right)}{m}-\frac{1}{3}\cdot \frac{\sum_{i}\text{std}\left(I_{i}\right)}{m}, \end{array}$$

where *I*_*i*_  (*i* = 1,…, *m*) denotes sources images needed to fuse and mean() denotes expectation function; std() denotes variance function; and the fired times can be computed as follows:

(7) $$\begin{array}{c} T_{ij}\left(\;n\;\right)=T_{ij}\left(\;n-1\;\right)+Y_{ij}\left(\;n\;\right), \end{array}$$

where *T*_*ij*_(*n*) denotes the total number of the fired times of neurons after the current iteration.

## 3. Image Fusion Based on RGF and SCM

Without loss of generality, we suppose that *A* and *B* are two medical images with different sensor to fuse, and *F* is the fused image.

Firstly, the RGF coefficients of *A* and *B* can be represented as follows. Note that all input images must be registered and also have the same size and identical resolution. Consider

(8) ARGFRGFA,BRGF=RGFB,

where RGF() denotes the RGF function.

Secondly, the normalized RGF coefficients are taken as the stimulus of the two SCMs to obtain

(9) TASCMARGF,TB=SCMBRGF,

where SCM() denotes the SCM with adaptive threshold *h* by (3)–(7). *T*_*A*_ and *T*_*B*_ denote the total fired times motivated by RGF coefficients *A*_RGF_ and *B*_RGF_, respectively.

Finally, the fused image *F* can be refined as follows:

(10) $$\begin{array}{c} F\left(\;i,j\;\right)=\left\{\begin{array}{cc} A\left(i,j\right) & \text{if }T_{A}-T_{B}>Tth\\ \frac{\left(A\left(i,j\right)+B\left(i,j\right)\right)}{2} & \text{if }\left|T_{A}-T_{B}\right|=Tth\\ B\left(i,j\right) & \text{otherwise.} \end{array}\right. \end{array}$$

**Figure 3:**
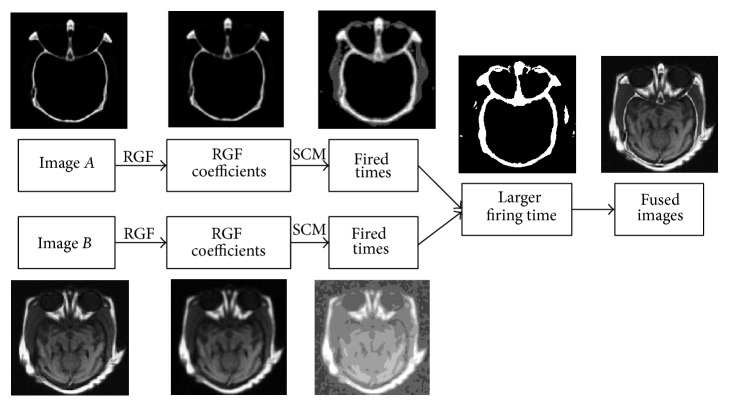
Schematic diagrams of fused image based on RGF and SCM.

In conclusion, the framework of the proposed fusion algorithm is shown in Figure 3.

## 4. Experimental Results

### 4.1. The Comparison of Other Fusion Methods

In order to evaluate the performance of the proposed fusion method, we introduce some objective criteria such as mutual information (MI) [8], *Q* ^*AB*/*F*^ metric [14], *L* ^*AB*/*F*^ metric [14], and *N* ^*AB*/*F*^ metric [14]. MI measures the amount of information transferred to the fused image from the source images. *Q* ^*AB*/*F*^ utilizes Sobel edge detector to measure the amount of edge information which is transferred from the source images to the fused image. In general, the higher MI and *Q* ^*AB*/*F*^ values indicate the better fused result. *L* ^*AB*/*F*^ is introduced to evaluate the information lost during the fusion process. The lost information is available in the source images but not in the fused image. *N* ^*AB*/*F*^ represents fusion artifacts that were introduced into the fused image. It is clear that the smaller *L* ^*AB*/*F*^ and *N* ^*AB*/*F*^ the better the fused image. It is worth noting that the complimentary *Q* ^*AB*/*F*^, *L* ^*AB*/*F*^, and *N* ^*AB*/*F*^ indicate that the sum of all these should result in unity [14]. Furthermore, the fused algorithms are evaluated by using the Matlab codes on Intel Core2 2.6 GHz machines with a 4 GB RAM.

**Figure 4:**
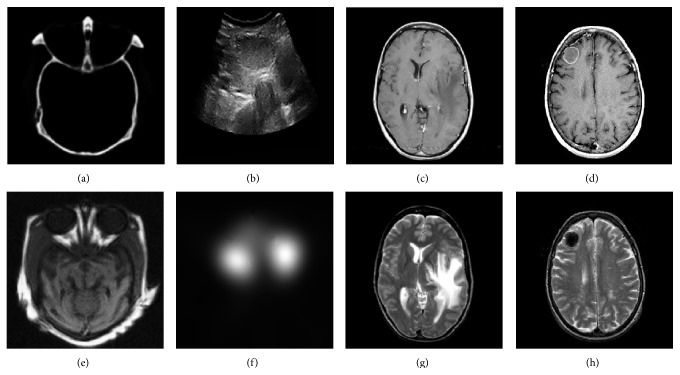
Different medical images to fuse. (a and e) show CT and MRI images of brain. (b and f) show B ultrasound and SPECT images of thyroid tumor. (c and g) show CT and MRI images of several focal lesions. (d and h) show T1-MRI and T2-MRI images that involved the lesion in the frontal lobe.

To evaluate the performance of the proposed fusion method, the experiments have been performed on four pairs of multimodal medical images as shown in Figure 4. These pairs of images are divided into the four groups. Group a contains Figures 4(a) and 4(e), and Figure 4(a) is a CT image of brain which can provide information of bones.  Figure 4(e) is an MRI image of brain which can provide some information about soft tissue. Group b contains Figures 4(b) and 4(f), and Figure 4(b) is B ultrasound image of thyroid tumor, as the anatomical imaging, providing organ organization structure information.  Figure 4(f) is SPECT image of thyroid tumors, as functional imaging, which can provide information of benignancy and malignanancy of thyroid tumor. Group c contains Figures 4(c) and 4(g), and they are CT image and T1-weighted MR-GAD image of several focal lesions involving basal ganglia. Group d contains Figures 4(d) and 4(h), and they are T1-MRI and T2-MRI that involve the lesion in the frontal lobe.

The following algorithms are used for comparison studies in the experiments: (1) GFF based on guided filtering proposed in [5] (GFF), (2) medical image fusion based on nonsubsampled direction complex wavelet transform proposed in [7] (NDCWT), (3) NSCT-based multimodal medical image fusion using pulse-coupled neural network and modified spatial frequency proposed in [8] (NSCT-SF-PCNN), (4) fusion algorithm based on Shearlet transform and PCNN proposed in [10] (ST-PCNN), and (5) fusion algorithm based on SCM proposed in [11] (SCM). For fair comparison, we use the parameters that were reported by the authors to yield the best fusion results. In our method, *σ*_*r*_ = 0.05 and *σ*_*s*_ = 1.2, and the iteration number of RGF is set to 4. The parameters of SCM are set as follows: *g* = 0.7, *f* = 0.8; synaptic linking weight *W* = [0.1091, 0.1409, 0.1091; 0.1409, 0, 0.1409; 0.1091, 0.1409, 0.1091]; iteration times *n* = 40 and the constant threshold *T*th = 1. To verify the effect of each part of our algorithm, fused method based on SCM with self-adaptive threshold *h* (SCM-A) and fused method based on RGF and SCM with constant threshold *h* (RGF-SCM-C) are also compared to our method (RGF-SCM).

**Figure 5:**
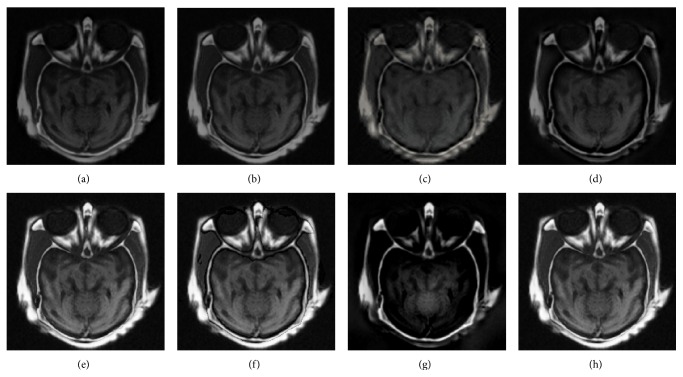
Fusion results of group a. (a–h) are the fusion images by GFF, NDCWT, NSCT-SF-PCNN, ST-PCNN, SCM, SCM-A, RGF-SCM-C, and RGF-SCM.

**Figure 6:**
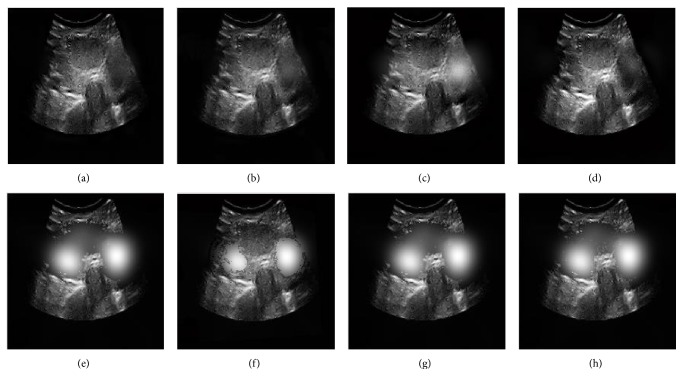
Fusion results of group b. (a–h) are the fusion images by GFF, NDCWT, NSCT-SF-PCNN, ST-PCNN, SCM, SCM-A, RGF-SCM-C, and RGF-SCM.

**Figure 7:**
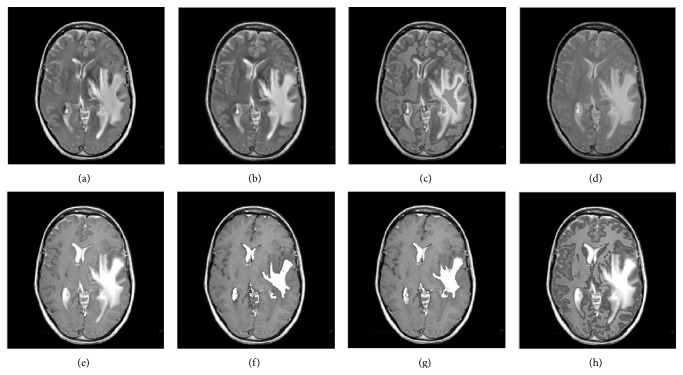
Fusion results of group c. (a–h) are the fusion images by GFF, NDCWT, NSCT-SF-PCNN, ST-PCNN, SCM, SCM-A, RGF-SCM-C, and RGF-SCM.

**Figure 8:**
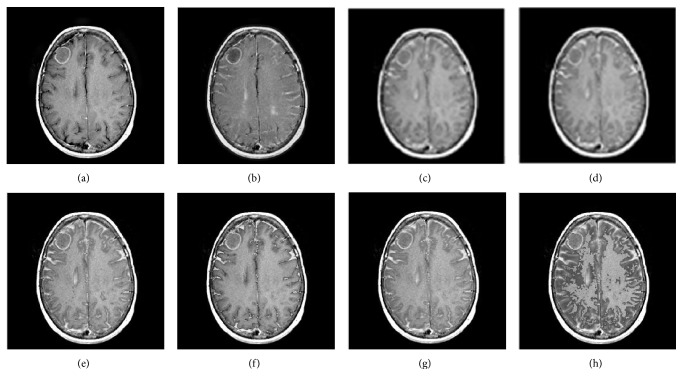
Fusion results of group d. (a–h) are the fusion images by GFF, NDCWT, NSCT-SF-PCNN, ST-PCNN, SCM, SCM-A, RGF-SCM-C, and RGF-SCM.

The above methods are utilized to fuse four group images in Figure 4, respectively. Figures 5–8 show the fused images by eight fused methods. From the fusion results in Figures 5(a)–5(h), it can be clearly seen that the image fused by our method reaches a higher contrast among all the fused images. Comparing the fused images of each algorithm, we can see that the proposed fusion algorithm preserves the texture information of source images well at the upright of the fused image. At the same time, less useless image information such as block effect and artifacts are introduced in the fused images in present scheme.

The fusion results of the eight algorithms in Figures 6(a)–6(h) show that our method has the best visual effect in all the fused methods. Figures 6(a)–6(d) show that GFF, NDCWT, NSCT-SF-PCNN, and ST-PCNN cannot fuse this type of medical images well; and Figures 6(e)–6(h) show that the method based on SCM can achieve much better performances. Comparing the fused image of SCM, our method not only preserves the texture information of source images but also suppresses useless image information such as block effect and artifacts, which should be attributed to the adaptive threshold in SCM and the saliency of medical images which is captured by RGF.

From the objective criteria shown in Table 1, one can find that our algorithm has the best objective criteria. The highest MI and *Q* ^*AB*/*F*^ mean that most useful information and edge information are converted into the fused result by our algorithm. The least *L* ^*AB*/*F*^ means that fewest information of source images is lost by our method. The least *N* ^*AB*/*F*^ means that least fusion artifacts are introduced into the fused image by our method. Therefore, our method can be regarded as a kind of good medical image fusion algorithm.

Figures 7 and 8 show the fused images of group c and d by eight fused methods. The fusion results shown in Figures 7(a)–7(f) and 8(a)–8(f) indicate that our method both has a higher contrast in all the fused methods and preserves the texture information of source images, suppressing useless image information such as block effect and artifacts.

From the objective criteria shown in Table 2, we can find that our algorithm always has the best objective criteria. Therefore, our method can be regarded as a robust medical image fusion algorithm.

### 4.2. The Robust to Noise

In order to validate the robustness of the algorithm, Gaussian noise with different noise variance from 5 to 50 is added to group a. The peak signal to noise ratio (PSNR) [15] is used to evaluate the performance of different fused methods. As the perfect fused image does not exist, the average of PSNR between fused image and source images is computed as measurement. It is defined as follows:

(11) $$\begin{array}{c} PSNR=\frac{\sum_{i}^{m}PSNR\left(I_{i},F\right)}{m}, \end{array}$$

where *I*_*i*_  (*i* = 1,…, *m*) denotes source images needed to fuse. *F* denotes the fused image.

**Figure 9:**
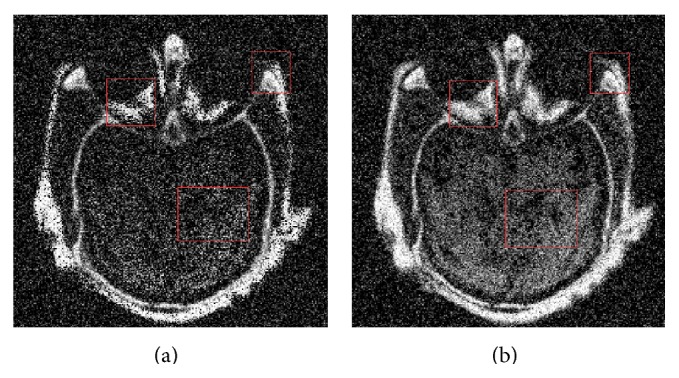
Fusion results of group a with noise variance being 35. (a) is the fusion image by SCM. (b) is the fusion image by RGF-SCM.

Figure 9(a) shows the fused image by SCM and Figure 9(b) shows the fused image by RGF-SCM. Obviously, our method has better visual performance than SCM.

**Figure 10:**
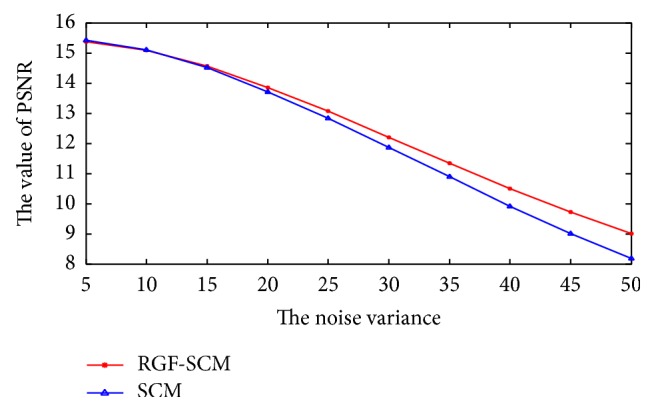
The PSNR of fused images by SCM and RGF-SCM with different noise variance. The PSNR of fused image by RGF-SCM is higher than SCM when the noise variance is grown.

Figure 10 shows PSNR of fused images by RGF-SCM and SCM. Obviously, the PSNR of fused image by RGF-SCM is higher than that by SCM when the source images have heavy noises. When the noise variance increases, the difference between PSNR of fused images by RGF-SCM and SCM increases too. It means that the performance of RGF-SCM becomes more efficient than SCM when the noise variance grows. Therefore, our method can be regarded as a robust medical image fusion algorithm.

## 5. Conclusions

A new fused method based on RGF and improved SCM is proposed to improve the medical fusion effect. The new fused method can enhance robustness to noise and extend SCM to fuse other kinds of medical images. Experimental results demonstrate that the proposed method is better than state-of-the-art medical image fusion methods in both visual appearance and objective criteria. In this paper, we just only cover the fusion of 2D images; however, 3D data sets are becoming increasingly important in medical procedure. It would be interesting to know whether and how an application to 3D data sets could be achieved. In the future research, we will extend current work to 3D data sets.

## Figures and Tables

**Table 1:** Objective criteria by each fused method in the fusion of Figures 5 and 6.

Fusion method	Group a				Group b			
	MI	*Q* ^*AB*/*F*^	*L* ^*AB*/*F*^	*N* ^*AB*/*F*^	MI	*Q* ^*AB*/*F*^	*L* ^*AB*/*F*^	*N* ^*AB*/*F*^
GFF	3.4313	0.7789	0.1405	0.0806	3.4736	0.7649	0.1698	0.0653
NDCWT	4.3905	0.7555	0.1401	0.1038	3.3573	0.7424	0.1759	0.0781
NSCT-SF-PCNN	4.8300	0.3635	0.1480	0.4885	3.1594	0.7073	0.1875	0.1052
ST-PCNN	2.2828	0.6761	0.2175	0.1064	4.9266	0.7627	0.1854	0.0519
SCM	6.0768	0.8603	0.1350	0.0046	**6.1210**	0.8203	0.1705	0.0091
SCM-A	6.0801	0.8610	0.1347	0.0043	**6.1210**	0.8254	0.1667	0.0079
RGF-SCM-C	6.0887	0.8624	0.1343	0.0033	6.1208	0.8274	0.1665	**0.0061**
RGF-SCM	**6.0947**	**0.8636**	**0.1333**	**0.0031**	**6.1210**	**0.8327**	**0.1612**	**0.0061**

**Table 2:** Objective criteria by each fused method in the fusion of Figures 7 and 8.

Fusion method	Group c				Group d			
	MI	*Q* ^*AB*/*F*^	*L* ^*AB*/*F*^	*N* ^*AB*/*F*^	MI	*Q* ^*AB*/*F*^	*L* ^*AB*/*F*^	*N* ^*AB*/*F*^
GFF	3.4548	0.8217	0.1358	0.0425	4.1974	0.7875	0.2089	0.0036
NDCWT	3.4854	0.7037	0.1973	0.0990	3.2677	0.5641	0.2561	0.1798
NSCT-SF-PCNN	3.4376	0.7486	0.2236	0.0278	4.0588	0.5710	0.2874	0.0216
ST-PCNN	4.3719	0.7157	0.2354	0.0489	4.1847	0.7740	0.2090	0.0170
SCM	5.6808	0.8360	0.1277	0.0363	5.5797	0.7979	0.1928	0.0093
SCM-A	5.6913	0.8277	0.1528	0.0195	5.5910	0.7983	0.1922	0.0095
RGF-SCM-C	5.7396	0.8475	0.1269	0.0256	5.6031	0.7988	0.1920	**0.0092**
RGF-SCM	**5.8156**	**0.8525**	**0.1265**	**0.0210**	**5.6414**	**0.8020**	**0.1888**	**0.0092**
